# Population genetics of *Todarodes pacificus* (Cephalopoda: Ommastrephidae) in the northwest Pacific Ocean via GBS sequencing

**DOI:** 10.1515/biol-2022-0876

**Published:** 2024-06-27

**Authors:** Yang Liu, Xingxing Hu, Huajie Lu, Yimeng Liu, Congcong Wang, Xiaojie Dai

**Affiliations:** College of Marine Living Resource Sciences and Management, Shanghai Ocean University, Shanghai, 201306, China; Marine Biomedical Science and Technology Innovation Platform of Lin-gang Special Area, Ministry of Agriculture and Rural Affairs, Shanghai, 201306, P.R. China; Scientific Research Department, Qingdao Technical College, Qingdao, Shandong, 266229, P.R. China; National Distant-Water Fisheries Engineering Research Center, Shanghai Ocean University, Shanghai, 201306, P.R. China; Key Laboratory of Sustainable Exploitation of Oceanic Fisheries Resources, Ministry of Education, Shanghai, 201306, P.R. China

**Keywords:** *Todarodes pacificus*, genotyping-by-sequencing, SNPs, population genetics

## Abstract

The common squid, *Todarodes pacificus*, is an important commercial species that inhabits the northwest Pacific Ocean, particularly the East Japan Sea, the Pacific coast of Japan, and the East China Sea. In this study, we chose 29 individuals from three areas: one type from the Sea of Japan and two types from the East China Sea. A total of 43,529 SNPs were obtained using genotyping-by-sequencing (GBS). Our analyses revealed low genetic diversity and genetic differentiation in each type. Heterozygote deficiency and inbreeding have caused this low level of genetic diversity. Population structure analysis indicated that the three types were genetically similar, which may be attributed to strong gene flow combined with the demographic history events during the last 2 million years. This new GBS application technique provides valuable information for the conservation of marine species genetics and could be useful for the effective management of this resource.

## 1 Introduction

*Todarodes pacificus* is a warm ocean cephalopod from the Ommastrephidae family that mainly lives in the northwest region (20°–60°N) of the Pacific Ocean. The primary fishing grounds for *T. pacificus* are distributed in the Sea of Japan and the East China Sea [1–3]. In some Asian countries, *T. pacificus* is a widely consumed aquatic food because of its abundant protein and microelement content [4]. In Japan, its production reached 5,600 tons in 2016, and it has become a primary target species of the squid industry in China [5,6]. It is one of the earliest developed and utilized species worldwide, with annual catches thereof accounting for approximately 60% of the total amount of cephalopods, which is the second largest mass-processed product worldwide [7]. Because of its short 1-year lifespan, the biomass of *T. pacificus* fluctuates widely under different biological and physical conditions [8,9]. Considering the spawning seasons, *T. pacificus* can be grouped into three groups (summer, autumn, and winter) [10]. To understand the population history, it is beneficial to evaluate the *T. pacificus* population structure and genetic diversity to obtain a high-resolution profile.

A previous study that focus on squid have been limited to utilizing fishery production statistics for forecast models to assess the variation in resource abundance [11]. Traditional morphological characteristics were the initial markers used to study phylogenetic relationships between and within populations before the development of molecular markers [12,13]. However, some problems could not be solved, and disagreements remained regarding phylogenetic matters. For instance, it was challenging to precisely distinguish the relationships within the Todarodinae and Ommastrephinae subfamilies using morphology alone [14]. The development of molecular markers has progressed over several decades from secondary metabolites to enzyme and DNA markers and finally to targeted sequencing techniques, which include microsatellite, genotyping-by-sequencing (GBS), and restriction fragment length polymorphism analyses. Molecular markers have become powerful tools in the field of genetic research. However, a limited number of molecular biology studies have been conducted on *T. pacificus*, such as the isolation of 21 polymorphic microsatellite loci from a size-selected genomic library [15], the isolation of 11 polymorphic microsatellite loci from *T. pacificus* [16], and the determination of the mitochondrial genomes of *T. pacificus* [17].

A single nucleotide polymorphism (SNP) refers to the substitution, deletion, or insertion of a single nucleotide that has occurred within the genome. They are generally located in a noncoding region of the genome and can be related to diseases or different phenotypes [18]. With the rapid development of next-generation sequencing (NGS), high-throughput SNP assays can be performed by sequencing short lengths across the whole genome. This reduces the complexity, increases the efficiency, and saves time on sample analysis when compared to older technologies [19,20]. GBS is a technology that can reduce the complexity of large genome sequence analysis by only sequencing the regions that are digested by specific restriction enzymes. This approach is suitable for the analysis of species with a large or incomplete genome. Additionally, GBS requires a lower amount of input DNA (≥800 ng) to perform the simple procedure, which is extremely specific and highly reproducible [21].

In this study, the GBS technique was utilized to identify several SNPs in the genome of *T. pacificus* and to analyze the population structure, genetic diversity, and historical dynamics of three groups collected from the Sea of Japan and the East China Sea. Our findings will help elucidate the historical events that impacted the demographics of *T. pacificus* and assist with the development of a genetic conservation strategy.

## 2 Materials and methods

### 2.1 Animal samples and DNA extraction

Twenty-nine samples were collected from three locations: (1) Sea of Japan (JS; *N* = 10; 36°11′N, 131°15′E) in January 2019; (2) East China Sea collection site 1 (ES1; *N* = 10; 27°16′N, 132°32′E) in June 2018; and (3) East China Sea collection site 2 (ES2; *N* = 9; 25°59′N, 122°28′E) in December 2018. The samples were randomly collected from the above sea waters.

The genomic DNA was extracted from approximately 25 mg of muscle using a modified phenol–chloroform procedure, following the protocol described by Gu and Xu [22]. Additionally, the genomic DNA was digested for 1 h with RNase A at 37°C (Takara, Japan) to remove the RNA. DNA purity and concentration were determined using a NanoDrop 2000 (Thermo Fisher Scientific, USA) spectrophotometer to obtain the optical density (OD) ratios of OD_260/280_ and OD_260/230_. Subsequently, 1% agarose gel electrophoresis was performed to assess the integrity of the genomic DNA. A minimum of 800 ng of DNA was used to create the library.


**Ethical approval:** The research related to animal use has been complied with all the relevant national regulations and institutional policies for the care and use of animals.

### 2.2 GBS library preparation and sequencing

The GBS libraries were prepared using two restriction enzymes (*Taq* I and *Mse* I; New England Biolabs, USA) to digest the double-stranded genomic DNA into several fragments with a size range of 500–600 bp. A sample-specific adapter was ligated to the end of the digested fragments, which were then pooled together. The size selection system was used to filter 450–700 bp fragments from the pooled mixture. The target GBS tags were amplified with specific primers to produce a final 400 bp product for sequencing on the Illumina Hiseq^TM^, paired-end 150. The above steps were conducted at Majorbio Pharm Technology Co., Ltd, Shanghai.

### 2.3 Bioinformatics and SNP genotyping

The raw GBSseq Illumina sequencing reads were converted to the FASTQ format using the Base Calling program. The program “process_radtags” v.2.26 was used to check the barcodes and demultiplex the sequencing data obtained from the Illumina platform. We removed the adapter sequences and discarded the bases with low average quality scores (quality value 10) using the Stacks 2.3 pipeline.

A set of high-quality short reads were aligned and grouped into their loci according to the depth parameters: (a) minimum depth of coverage (−m) of five and (b) maximum distance allowed of two. The cstacks program was used to establish a catalog of the loci in which the alleles were merged, and a consensus was produced, called “stacks.” All of the samples were matched against the stacks, and the allele status was determined using stacks [23]. The SNPs were identified using the putative loci as well as the maximum likelihood framework. They were further screened according to the following parameters to obtain high-quality SNPs: minor allowed frequency ≥ 0.05, missing ratio ≤ 0.2, and the lowest depth = 2.

### 2.4 Data analysis

#### 2.4.1 Genetic diversity

The population genetic diversity statistics, which included the private SNP number (Private), observed heterozygosity (Obs_He), expected heterozygosity (Exp_He), observed homozygosity (Obs_Ho), expected homozygosity (Exp_Ho), nucleotide diversity (Pi), population coefficient of inbreeding (Fis), and the genetic differentiation index (Fst), were calculated [24]. Fis was used to assess whether the existing inbreeding generates a hidden population and leads to a reduction in heterozygosity [25]. Fst is an important parameter to measure the degree of genetic differentiation among populations.

#### 2.4.2 Phylogenetic tree analysis

To assess the phylogenetic relationship between samples from the three different regions, RAxML version 8 was used for the phylogenetic analysis. The genetic relationship between the samples was calculated and represented using a tree-like diagram [26]. The analysis was based on SNPs and the maximum likelihood algorithm.

#### 2.4.3 Population structure analysis

For the population structure investigation, we utilized a freely available software program, ADMIXTURE 1.3.0 (http://www.genetics.ucla.edu/software/admixture/). This program depends on the model-based Bayesian analysis that uses the loci of the filtered SNP. Briefly, the number of subpopulations (*k*) of samples was estimated to be within the range of 2–19, and the optimal *k* value was determined based on the cross-validation error (CV error), where the lowest CV error value corresponds to the optimal *k* [27].

#### 2.4.4 Principal component analysis (PCA)

We performed PCA to assess the proportion of phenotypic variance using the GCTA 1.26.0 software [28]. The 29 samples were clustered according to complex traits using the SNPs, and a mathematical method [29].

#### 2.4.5 Population history

The study of the population history included effective population size, gene flow, and differentiation time. Effective population size was estimated using Pairwise Sequentially Markovian Coalescent, an R software package, which is a statistic that describes the density variance of the heterozygous SNP loci in the different regions of the genome. Population differentiation time was calculated using Beast2 [30], which analyzes the molecular sequence phylogeny conditions based on Bayes evolutionary theory. The Markov Chain Monte Carlo algorithm was used to obtain the average space of the tree [31], and the Beauti and TreeAnnotator programs were used to calculate the divergence time.

## 3 Results and discussion

### 3.1 GBS and data generation

Currently, NGS-based SNP methods are widely used in many fields, such as genetic diversity estimation [32], population, and evolutionary studies [33]. To date, most studies have focused on changes in *T. pacificus* abundance or fisheries hydrography. However, no studies have used GBS for population genetics analysis or resource stock assessments of this species in the Sea of Japan and the East China Sea. Twenty-one polymorphic microsatellite loci were detected in a *T. pacificus* population from Korean waters and were used to estimate the genetic diversity and population structure. However, of the 31 primer sets used for PCR, only 21 were successful, and the degree of loci cover was only 67.7%. The accuracy of this genetic data should be examined further. The identification of highly polymorphic genetic markers for the accurate analysis of genetic composition and differentiation is vital for the study of natural populations of *T. pacificus*.

**Figure 1:**
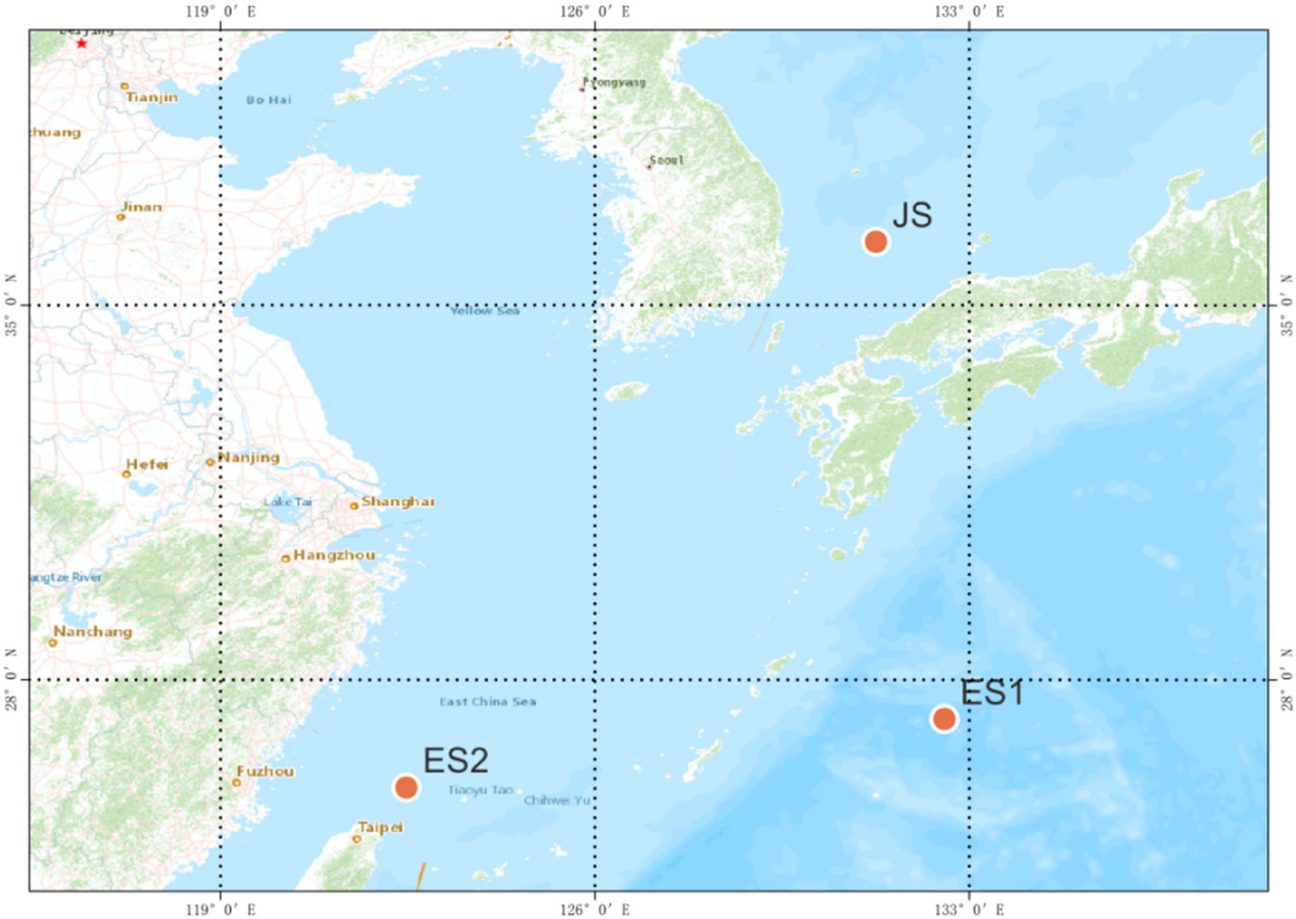
Geographical location of *T. pacificus* population samples analyzed in this study. The red dots indicate the areas of the Sea of Japan (JS), the East China Sea collection site 1 (ES1), and the East China Sea collection site 2 (ES2).

In this study, the 29 collected samples were divided into three subgroups in accordance with the sampling areas and were named JS, ES1, and ES2. The sampling site for each subgroup is shown in Figure 1. Illumina sequencing yielded a total of 140.59 GB of raw data, with an average of 4.85 GB for each sample. The base quality, Q30, was obtained by 91.99% of the reads, and the average depth achieved was 16.88× (Table S1). We generated abundant, high-quality, clean reads through stringent quality control, which was implemented using several programs included in the Stacks 2.3 Pipeline. A total of 8,589,120 SNPs were identified and genotyped in the 29 samples. After the tag filter was used with the standard parameters, 43,529 high-quality SNP markers remained and were used for further analysis and determination of the population evolution.

In this study, a large number of high-quality SNPs were generated for *T. pacificus*, which will prove effective for assessing the genetic structure of this species from the Pacific Ocean. This genotyping technique may serve as a valuable exploration tool for the development of high-throughput SNPs and for performing genetic divergence studies in marine species.

### 3.2 Population genetic diversity

The estimated genetic diversity across the three sample types is summarized in Table 1. The mean values of the observed and expected heterozygosity were 0.1175 (range 0.11444–0.12168) and 0.1664 (range 0.16147–0.17145), respectively. This demonstrates that the average Obs_He in the three types was relatively low and that the Exp_He was slightly higher. This indicates that the genetic diversity of *T. pacificus* in the Sea of Japan and the East China Sea has decreased. It has been demonstrated that overfishing results in the decline of genetic diversity in marine fish, and it is possible that the overexploitation of *T. pacificus* in the 1870s and 1880s, which resulted in a drastic reduction of the overall stock, may have contributed to the low haplotypic diversity of *T. pacificus* as well as the general low genetic diversity of *T. pacificus* [1]. The Fis values were positive, ranging from 0.17806 to 0.1992, which indicates that inbreeding may have occurred within the types. Random amplified polymorphic DNA and microsatellite analyses have identified low diversity in other Cephalopoda squid (*Dosidicus gigas*) [34,35]. However, high levels of observed (range 0.417–0.917) and expected (range 0.791–0.965) heterozygosity were reported in the Korean *T. pacificus* populations, as demonstrated by microsatellite analysis of the genetic diversity and structure [36]. *T. pacificus* is a migratory organism and environmental and ocean current factors markedly affect its growth and reproduction processes. The low levels of diversity in *T. pacificus* within the Sea of Japan and the East China Sea might be attributed to the high migration capacity of the spawning group and the demographic expansion between the glacial and interglacial types.

**Table 1:** Statistical values of the genetic diversity of the three populations

Population	Private	Num_Indv	Obs_He	Obs_Ho	Exp_He	Exp_Ho	Pi	Fis
ES1	1,711,590	3.12905	0.1163	0.8837	0.17145	0.82855	0.22254	0.1992
JS	1,586,434	2.95875	0.12168	0.87832	0.16629	0.83371	0.21885	0.17806
ES2	1,506,149	2.86326	0.11444	0.88556	0.16147	0.83853	0.21243	0.17964

Note: Num_Indv, Obs_Het, observed heterozygosity; Obs_Hom, observed homozygosity; Exp_Het, expected heterozygosity; Exp_Hom, expected homozygosity; Pi, nucleotide diversity; Fis, inbreeding coefficient.

### 3.3 Population genetic differentiation

**Figure 2:**
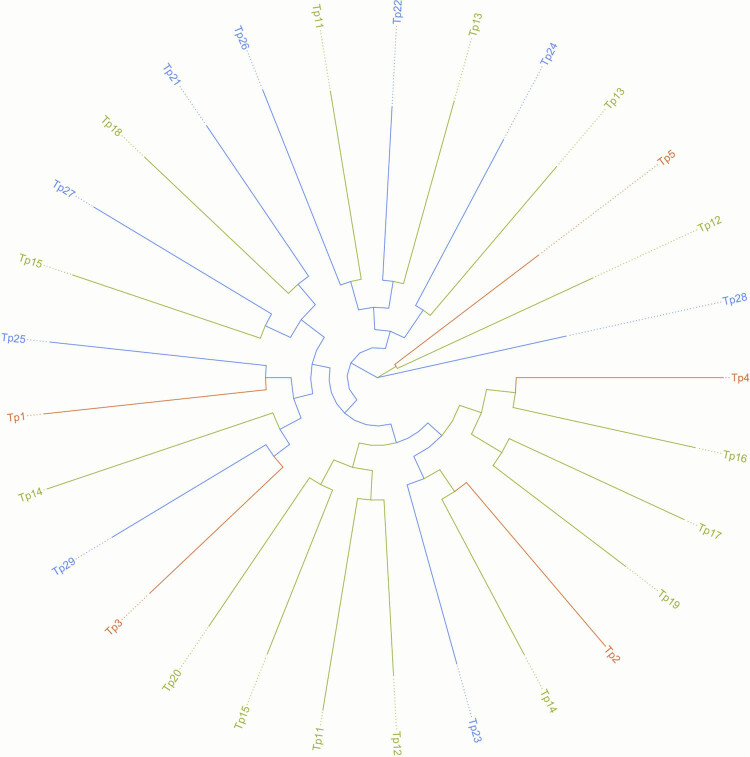
NJ tree for *T. pacificus* constructed using the RAxML program based on the high-quality SNPs. Brown represents the East China Sea collection site 1 (ES1) type, green represents the Sea of Japan (JS) type, and blue represents the East China Sea collection site 2 (ES2) type.

To analyze the population structure of the three types, a neighbor-joining (NJ) tree was constructed using the RAxML program. Figure 2 shows the phylogenetic relationship between the 29 individuals. Each branch represents an individual, and the root of the tree indicates a possible ancestor. A mixed NJ tree revealed that the 29 individuals from the three different regions were not clearly divided or clustered but were rather intermingled across the tree. This result demonstrates that the *T. pacificus* caught at the three sites did not show significant genetic differentiation.

**Figure 3:**
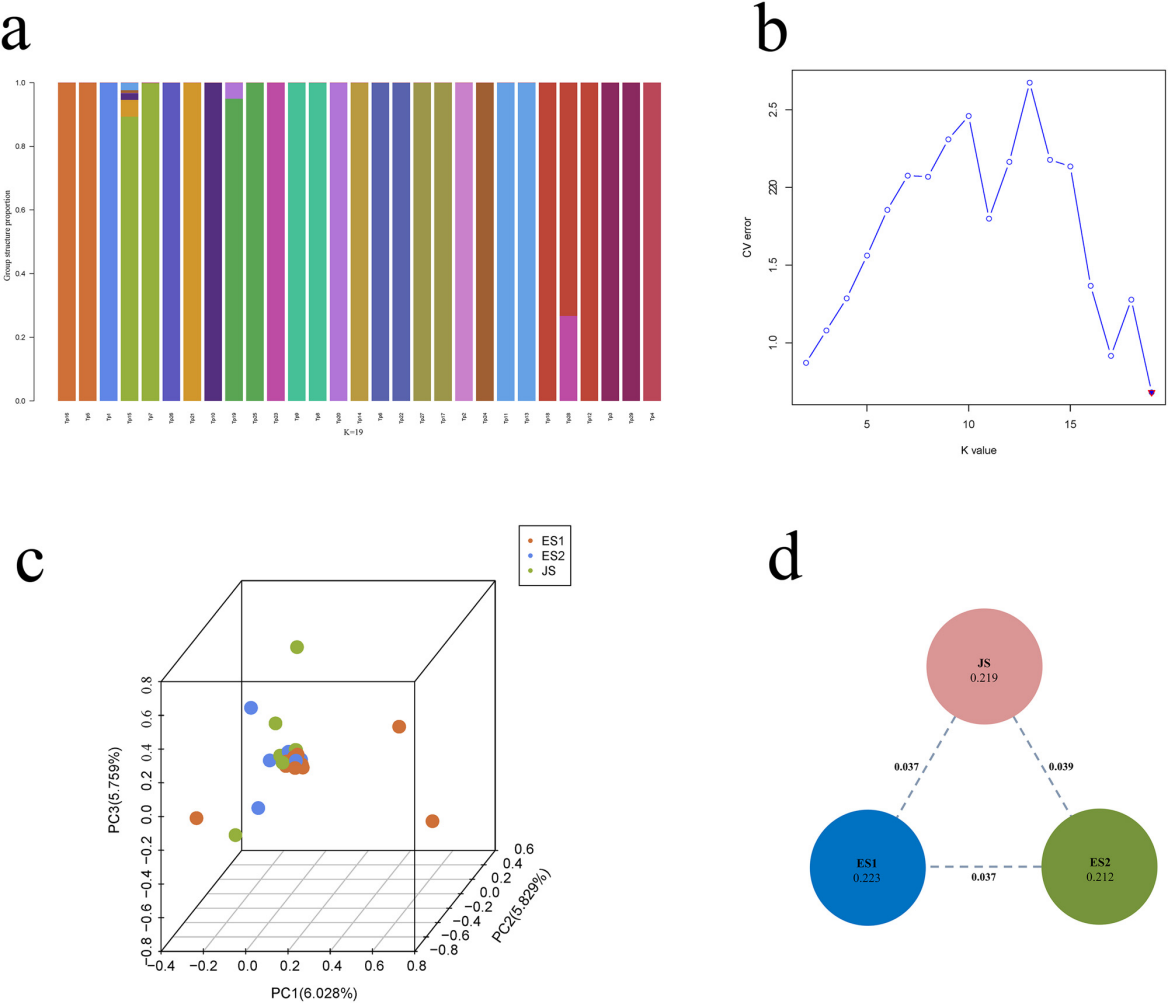
Population genetic differentiation analysis of *T. pacificus.* (a) The *Y* axis represents the putative ancestry and the *X*-axis shows the different individuals. Clustering of the predefined number of populations (*k*) from 2 to 19. (b) CV error. (c) PCA of the 29 individuals was performed using the GCTA program. Dots represent individuals, and colors denote the sampling origin: East China Sea collection site 1 (ES1: brown), Sea of Japan (JS: green), and East China Sea collection site 2 (ES2: blue). (d) Nucleotide diversity (Pi) and genetic differentiation (Fst) across the three sample types. The value in each circle represents a measure of Pi for this sample type, and the value on each line indicates genetic differentiation between the two types.

The population genetic differentiation index can describe the degree of differentiation between populations. The pairwise Fst values among the three types ranged from 0.0371 to 0.0393, which is lower than the medium differentiation index (Fst < 0.05) (Figure 3d). This suggests that the populations from the three sampling sites belong to a single population. This further suggests that no obvious genetic differentiation exists within any of the types. This result was similar to those previously generated using mitochondrial DNA markers [37].

Genetic structure was estimated using the ADMIXTURE program, with the putative values of *k* ranging from 2 to 19 based on the screening of tight linkage loci from several SNPs. The optimal number of *k* subpopulations was identified when the CV error reached the lowest point. The results revealed that the 29 individuals were linked to 19 ancestors (Figure 3a and b). The number of ancestors from JS, ES1, and ES2 was 9, 10, and 7, respectively. It was clear that the individual Tp10 originated from the ES1 region and possessed mixed ancestry, whereas individuals Tp15 and Tp17 were both originated from the JS site and developed from two distinct ancestries. PCA substantiated the population genetic distance based on the distance between the different components. Most of the samples gathered into one cluster, whereas several individuals were irregularly interspersed throughout the PCA. This suggests that the three sample types originate from different areas but have a close genetic relationship (Figure 3c). Based on these findings, it can be observed that there is no genetic differentiation among the three *T. pacificus* types.

The migratory routes of *T. pacificus* may overlap because of the wide fluctuations in the stocks and specific spawning groups (summer, autumn, and winter). Additionally, the instability of the biological and physical environments may also influence the population structure [38,39]. A reasonable explanation for these results could be that the northeast of the East China Sea is connected to the Sea of Japan by the Kuroshio and Tsushima warm currents. This sea area is strongly influenced by the ocean currents throughout the year, and ocean currents play a significant role in transportation. Therefore, the *T. pacificus* populations have no obvious geographical isolation [40]. Meanwhile, *T. pacificus* can lay eggs in batches in different seasons and can do so for a long time and in large quantities, where their high migratory capacity and pelagic egg traits may contribute to the strong gene flow across their geographical distribution and thereby reduce the genetic differentiation between the populations [41,42]. Incredibly strong gene flow, Nm > 1, was found in our unpublished study using mitochondrial DNA markers, which suggests that frequent genetic exchange could reduce genetic differentiation. Another possible cause for the low level of differentiation is fishing pressure, which can result in heterozygote removal.

### 3.4 Population history

**Figure 4:**
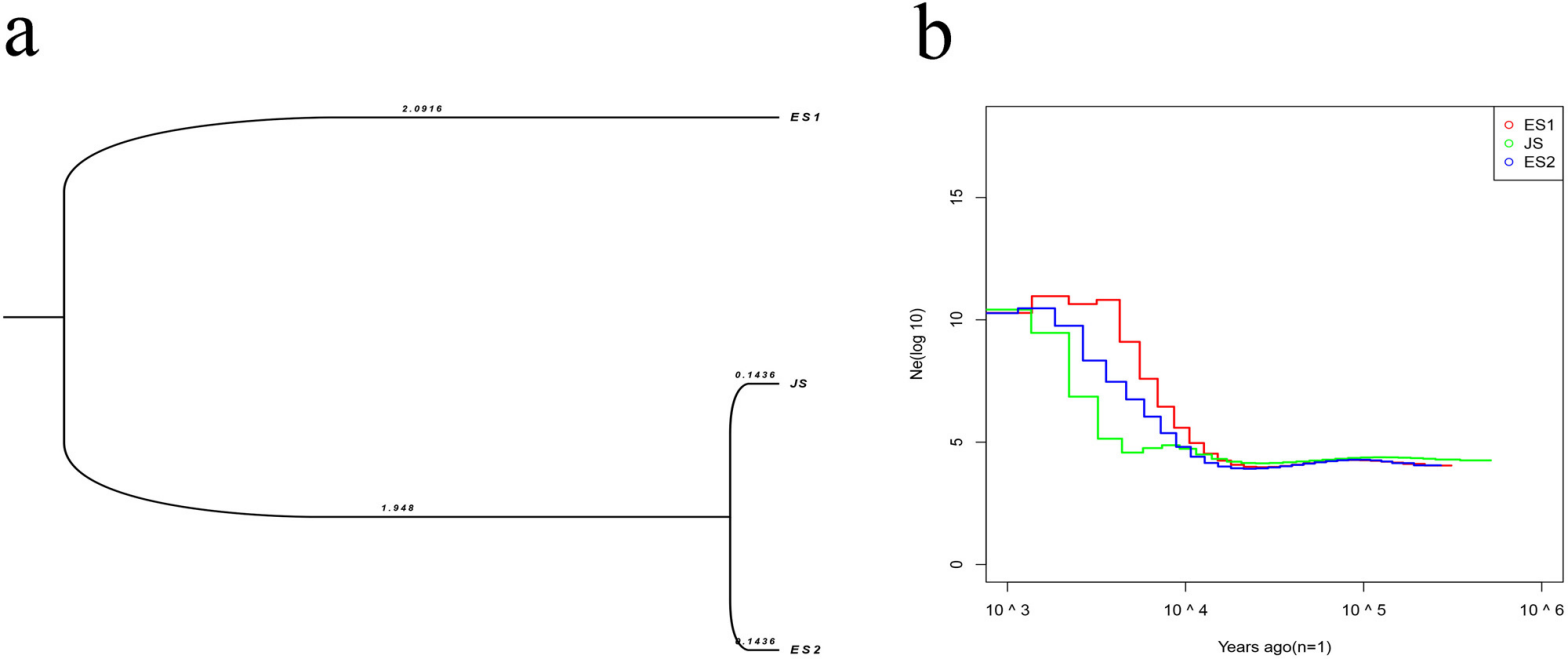
Population history analysis of *T. pacificus*. (a) The population differentiation time plot was based on a Bayesian phylogenetic analysis system. The number in the figure represents the divergence time, and the scale of the axes is measured in millions of years. (b) Estimation of the effective population size for the East China Sea collection site 1 (ES1), Sea of Japan (JS), and East China Sea collection site 2 (ES2) sample types. The curve represents changes in the effective population size, colors denote the different populations, and the *X*-axis indicates the timeline.

Analysis of historical population dynamics can be used to deduce the evolutionary characteristics of a population, such as divergence time, effective population size, and gene flow. In this study, we investigated the historical dynamic variation of the population. The effective population size (Ne) of the three sample types was maintained at a stable level until 100,000 years ago. A stepwise expansion, followed by a final steady state, was observed 10,000 years ago (Figure 4a). As a whole, the Ne in these three sample types presented an amplification phenomenon, which suggested that this population lived through a recent demographic expansion. The expansion time was estimated to be approximately 12,000–15,000 years ago, which corresponded to the late Pleistocene of the Quaternary ice ages (10,000–1,200,000 years ago) [43]. The different growth patterns of the *T. pacificus* populations in the Sea of Japan and the East China Sea may be the consequence of isolation between the two areas caused by a fall in sea level. To calculate the differentiation time between the related populations or the divergence time between the different populations, Beast2 was used to estimate the phylogeny. The differentiation time is shown in Figure 4b, and the JS and ES2 populations clustered in a branch that was established 1.948 million years ago and was then divided into two branches. The ES1 population separated from the JS and ES2 populations at least 2.0916 million years ago and underwent no further branching. Elevation of the sea levels from glacial ablation allowed the ES2 and JS types to be reconnected, resulting in strong gene flow 143,600 years ago. We speculate that the continual cycle between glaciation and interglaciation during the last 2 million years led to the current geographical pattern and differentiation as well as the genetic consequences within the three sample types [44].

## 4 Conclusion

In this study, the results from the GBS analysis provided the first description of the genetic diversity, population structure, and demographic history of *T. pacificus* populations present within the marginal sea of the northwest Pacific Ocean. The methodology showed that screening a large set of SNPs via GBS technology is a promising tool for evaluating the population genetics of migratory marine species. The relatively low level of genetic diversity and the weak genetic differentiation of *T. pacificus* around the Sea of Japan and the East China Sea may be attributed to a period of demographic expansion. An increased sample size over a larger geographic region is required to confirm whether *T. pacificus* populations from the two areas originate from the same population. A plan to manage quota allocations, catch size, and the age of *T. pacificus* around the Sea of Japan and East China Sea is necessary to prevent further loss of genetic diversity. Additionally, the integration of genetic data with resource status and environmental conditions will contribute toward the sustainable development of a conservation strategy for *T. pacificus*.

## Supplementary Material

Supplementary Table
