# Supplementary material for: Population genetics of Todarodes pacificus (Cephalopoda: Ommastrephidae) in the northwest Pacific Ocean via GBS sequencing
Source: Open Life Sci. 2024 Jun 27;19(1):20220876. doi: 10.1515/biol-2022-0876 (PMC11211871; doi:10.1515/biol-2022-0876)
Supplement: Supplementary Table [file biol-2022-0876-sm.pdf]

Supplementary material

Table S1: Sequencing parameters of the 29 samples

Sample ID	Clean reads	SNP number	Average depth	Q30 (%)
Tp1	11,656,771	539,043	13.79	91.95
Tp2	22,307,019	1,423,764	19.75	91.59
Tp3	20,324,770	256,400	17.82	91.56
Tp4	22,827,539	1,558,876	18.84	91.82
Tp5	17,445,782	231,958	16.57	91.7
Tp6	13,542,204	202,605	15.47	92.44
Tp7	15,221,551	215,562	16.27	91.82
Tp8	26,896,202	275,091	19.25	92.11
Tp9	8,823,896	803,554	13.8	91.9
Tp10	12,864,775	1,212,521	13.41	91.88
Tp11	16,099,457	1,416,391	14.63	92.12
Tp12	12,846,220	206,993	14.28	92.01
Tp13	10,589,198	173,773	15.01	91.87
Tp14	14,085,286	205,517	15.59	92.41
Tp15	15,844,786	207,850	17.17	91.64
Tp16	16,807,480	206,503	18.47	91.82
Tp17	22,893,922	215,547	23.11	91.6
Tp18	28,028,696	264,519	21.96	91.96
Tp19	10,321,949	155,231	16.22	91.86
Tp20	12,277,355	947,861	16.78	91.94
Tp21	16,772,188	240,872	15.32	92.15
Tp22	21,323,122	203,166	22.79	91.83
Tp23	19,109,142	245,075	16.38	92.61
Tp24	11,823,496	189,762	14.72	92.12
Tp25	16,682,912	226,543	16.55	92.64
Tp26	23,843,457	1,592,911	20.16	92.26
Tp27	20,032,014	249,233	17.02	92.17
Tp28	11,754,858	1,175,738	13.61	92.37
Tp29	13,327,901	214,513	14.67	91.93

Note: Q30, percentage of bases in the clean data with a quality value of ≥30.
